# Prevalence and Factors Associated with Anemia among Pregnant Women Attending Antenatal Clinic at St. Paul's Hospital Millennium Medical College, Addis Ababa, Ethiopia

**DOI:** 10.1155/2018/3942301

**Published:** 2018-08-29

**Authors:** Angesom Gebreweld, Aster Tsegaye

**Affiliations:** ^1^Department of Medical Laboratory Sciences, College of Medicine and Health Science, Wollo University, Dessie, Ethiopia; ^2^Department of Medical Laboratory Sciences, College of Health Science, Addis Ababa University, Ethiopia

## Abstract

**Background:**

In pregnancy, anemia is an important factor associated with an increased risk of maternal, fetal, and neonatal mortality, poor pregnancy outcomes, and impaired cognitive development, particularly in developing countries like Ethiopia. This study aimed to assess prevalence and factors associated with anemia among pregnant women attending antenatal clinic at St. Paul's Hospital Millennium Medical College, Addis Ababa, Ethiopia.

**Method:**

A cross-sectional health facility based study was conducted on 284 pregnant women to assess prevalence and factors associated with anemia at St. Paul's Hospital Millennium Medical College from June to August 2014. Data on sociodemographic and clinical characteristics of the study participants were collected using a pretested structured questionnaire by interview and review of medical records. About 4 ml of venous blood was collected from each subject for peripheral blood film and complete blood counts (CBC). Binary Logistic regression analysis had been used to check for association between dependent and independent variables. In all cases, P value less than 0.05 was considered statistically significant.

**Result:**

The prevalence of anemia was found to be 11.6% (95 % CI; 7.8%-14.8%). Pregnant women in the second [AOR (95% CI), 6.72 (1.17-38.45), and P=0.03] and third trimester [AOR (95% CI), 8.31 (1.24-55.45), and P=0.029] were more likely to be anemic when compared to pregnant women in their first trimester. Pregnant women who did not receive iron/folic acid supplementation [AOR (95%CI), 4.03(1.49-10.92), and P=0.01] were more likely to be anemic when compared to pregnant women who did take supplementations.

**Conclusion:**

In this study the prevalence of anemia in pregnancy was low compared to the findings of others. Gestational age (trimester) and iron/folic acid supplementation were statistically associated with anemia. Therefore, iron supplementation and health education to create awareness about the importance of early booking for antenatal care are recommended to reduce anemia.

## 1. Background

Anemia is a decrease in the oxygen carrying capacity of the blood. It can arise if the hemoglobin (Hb) concentration of the red blood cells (RBCs) or the packed cell volume of RBCs (PCV) is below the lower limit of the reference interval for the individual's age, gender, geographical location, and physiological status [1, 2].

During pregnancy the total blood volume increases by about 1.5 liter [3]. The plasma volume increases more compared to red cell mass which leading to hemodilution and reduced hemoglobin concentration. This is termed physiological anemia of pregnancy [3, 4]. The World Health Organization (WHO) has suggested that anemia is present in pregnancy when Hb level is <11g/dl. It also classified anemia in pregnancy as mild (10.0-10.9 g/dl), moderate (7.0-9.9 g/dl), and severe (lower than 7.0 g/dl) based on the level of hemoglobin concentration [5].

Anemia is a public health problem in both developed and developing countries. It affects 1.62 billion people globally, which corresponds to 24.8% of the world population. Global prevalence of anemia in pregnant women is 41.8% and the highest proportions of pregnant women affected are in Africa (57.1%) [6, 7]. According to Ethiopia Demographic and Health Survey report of 2005, the prevalence of anemia in pregnant women is 30.6% [8].

Approximately 50% of cases of anemia are considered to be due to iron deficiency. Anemia resulting from iron deficiency in pregnancy is an important factor associated with an increased risk of maternal, fetal, and neonatal mortality; poor pregnancy outcomes such as low birth weight and preterm birth; impaired cognitive development, reduced learning capacity, and diminished school performance in children; and decreased productivity in adults, particularly in developing countries like Ethiopia. In neighboring Sudan, 20.3% of maternal deaths are associated with anemia [9–11].

In Ethiopia, different studies were conducted on prevalence of anemia among pregnant women, the prevalence range being from 9.7% in North Shoa Zone to 56.8% in Eastern Ethiopia [12, 13]. Studying the specific etiology and prevalence of anemia in a given setting and population group is very important to prevent or treat anemia [11]. However, there is very little data available in the study area. Therefore, this study is aimed to assess the prevalence and factors associated with anemia in pregnant women at St. Paul's Hospital Millennium Medical College, Addis Ababa, Ethiopia.

## 2. Methods

### 2.1. Study Setting and Design

A facility based cross-sectional study was conducted from June to August 2014 at St. Paul's Hospital Millennium Medical College (SPHMMC), Addis Ababa, Ethiopia. SPHMMC was built in 1969 by Emperor Haile Selassie as a source of medical care for underserved populations. It currently has 392 beds, with an annual average of 200,000 patients and a catchment population of more than 5 million.

### 2.2. Study Population

All pregnant women attending antenatal cares in SPHMMC that fulfills the inclusion criteria during the study period were considered as study participants. Written informed consent was obtained from all. Pregnant women with Hepatitis B Virus infection, with human immunodeficiency virus, and less than 18 years of age were excluded from the study.

### 2.3. Sampling Procedure

The required sample size for this study was calculated using a single population proportion formula with a 95% CI, 5% margin of error, and assumption that 21.3 % of pregnant women are anemic [14]. By adding 10% for nonresponse, a total of 284 pregnant women were enrolled from antenatal care clinic of obstetrics and gynecology department of SPHMMC. Systematic random sampling technique was used to recruit the study participants from their sequence of ANC visit during the study period.

### 2.4. Data Collection

Interviewer administered structured pretested questionnaire and review of medical records were used to collect data on the sociodemographic characteristics, obstetric and gynecological, diet, and clinical characteristics of the study participants. The interview and record review were conducted by two trained ANC service provider nurses at ANC clinic of SPHMMC during ANC follow-up of the study participants. About 4 ml venous blood specimens were taken from each participant in K3-EDTA tubes for the hematological examinations. Automated hematology analyzer Cell-Dyn 1800 (Abbott Laboratories Diagnostics Division, USA) was used to determine complete blood count. Thin peripheral blood smears were prepared and stained by Wright's stain for red cells morphological study. Quality control materials were run alongside the study participant's sample to control performance of the hematological analyzer. All laboratory measurements were done by experienced laboratory technologists.

### 2.5. Data Analysis

Data from both questioner and laboratory were checked and cleaned for completeness and consistency. Data were then analyzed using Statistical Package for the Social Science (SPSS) Version 20 statistical software. Descriptive statistics such as frequency, percentage, and mean andstandard deviation were used to describe dependent and independent variables. Binary logistic regression analysis had been used to check for association between dependent and independent variables. In all cases P value less than 0.05 was considered statistically significant.

### 2.6. Ethical Considerations

Ethical clearance was obtained from both Research and Ethics Review Committee of the Department of Medical Laboratory Sciences, Addis Ababa University, and Institutional Review Board of SPHMMC. Written informed consent was obtained from each study participant after the purpose and importance of the study were explained. To ensure confidentiality, participants' data were linked to a code number. Any abnormal test results of participants were communicated to their attending physician.

## 3. Result

### 3.1. Characteristics of the Study Participants

A total of 284 pregnant women were included in the study. The mean age of the participants was 27.3 ± 4.5 years (range from 18-40). The majority of the study groups, 118 (41.5%), were in the age range of 26-30 years and 102 (35.9%) were in the weight group of 60-69 Kg. Most of the respondents, 261(91.9%), 164 (57.7%), and 115 (40.5%), were urban dwellers, house wives by occupation, and elementary school education level, respectively (Table 1).

Concerning obstetrical history and dietary habit, 170 (59.9%) were in their third trimester, 194 (68.3%) of the women had previous pregnancy, 166 (58.5%) were multigravida (2-4 pregnancy), 124 (43.7%) had no child, 28 (9.9%) had blood loss during the current pregnancy, 77 (27.1%) experienced abortion, and 166 (58.8%) had taken iron/folic acid supplementation. Out of the 284 participants, 160 (56.3%) had the habit of eating meat and animal products and 120 (42.3%) had the habit of eating fruit and vegetables once in week (Table 1).

### 3.2. Prevalence and Associated Risk Factors of Anemia

The mean ± SD hemoglobin concentration of the study participants was 13.0 ± 1.64 g/dl (ranges from 7.1-22.9 g/dl). In this study, the overall prevalence of anemia was 11.6% (95 % CI; 7.8%-14.8%) [15].

The rate of anemia was high in pregnant women who were in 26-30-year age range (15.3%), 60-69 weight group (17.6%), house wives (14.0%), elementary school (14.8%), and urban residents (12.6%) (Table 2).

Based on obstetric history and dietary habit, the prevalence of anemia was higher in pregnant women who were at the second trimester (16.7%), had previous history of pregnancy (12.4%), multigravida (12.7%), had one child (17.6%), had ≥ 4-year gap between the current and last child (16.7%), had history of abortion (14.3%), and did not take iron/folic acid supplementation (14.5%). The prevalence of anemia was also higher in those pregnant women who did not have a habit of eating meat and animal products (16.7%) and fruits and vegetables (33.3%) (Table 2).

All variables were analyzed using bivariate analysis to assess the association between the variables and anemia. Then, variables that show P value less than or equal to 0.3 in bivariate analysis were taken to multivariate analysis. Out of those variables treated under multivariate analysis, trimester and iron/folic acid supplementations were statistically significantly associated with anemia. Pregnant women in the second [AOR (95% CI), 6.72 (1.17-38.45), and P=0.03] and third trimester [AOR (95% CI), 8.31 (1.24-55.45), and P=0.029] were more likely to be anemic when compared to pregnant women in their first trimester. Pregnant women who did not receive iron/folic acid supplementation [AOR (95%CI), 4.03(1.49-10.92), and P=0.01] were more likely to be anemic when compared to pregnant women who did take supplementations.

## 4. Discussion

The prevalence of anemia in the present study was 11.6% (95 % CI; 7.8%-14.8%) [15]. This prevalence was almost consistent with studies conducted in Awassa (15.1%), Gondar (16.6%), Debre Berhan (9.7%), Sudan (10%), Iran (13.6%), and Nakhon Sawan, Thailand (14.1%) [12, 16–20]. However, our finding is much lower than studies conducted in Pakistan (90.5%), India (87.2%), Malaysia (57.4%), Benin (68.3%), Nigeria (54.5%), Somali Region (56.8%), Walayita Sodo (40%), West Arsi zone (36.6%), and north western zone of Tigray (36.1%) [13, 21–28]. Our result is also lower than results reported from Uganda (22.1%), Southern Ethiopia (29%), Southeast Ethiopia (27.9%) Mekelle (19.7%), and Addis Ababa (21.3%) [14, 29–32].

The difference may be due to geographical variation, differences in socioeconomic status, and dietary habits of the study participants. The lower finding of our study also may be due to the governments' effort to achieve Millennium Development Goals (MDGs) since improving maternal health is one of the eight MDGs and targeted to reduce the maternal mortality ratio by three-quarters in 2015. The United Nations Population Fund (UNFPA) report showed a significant decline in the prevalence of anemia among all women from 20% to 13% between 2005 and 2011 in Ethiopia [33].

Only the association of gestational age (trimester) and iron/folic acid supplementations did reach to a statistically significance level. Pregnant women in second and third trimester were more likely to be anemic when compared to pregnant women in first trimester. This might be due to the higher maternal plasma volume increments (40–50%) relative to red cell mass (20–30%) and accounts for the fall in hemoglobin concentration [34]. Our study is similar with studies conducted in Malaysia [22], west Algeria [35], and Tikur Anbessa hospital [14].

The risk of developing anemia increased in pregnant women who did not receive iron supplementation during pregnancy when compared to those who received iron supplementation. This may be due to iron deficiencies developing during pregnancy because of the increased iron requirements to supply the expanding blood volume of the mother and the rapidly growing fetus and placenta. The study is in agreement with study conducted in Karnataka India, Uganda, and Eastern Ethiopia [13, 36, 37].

One of the limitation of this study is the cross-sectional nature of the study design; it did not reveal causal links between anemia and risk factors. Even though this study tried to address some important factors, other factors such as stool examination, malaria, inherited, or acquired disorders that affect hemoglobin or red blood cell synthesis were not addressed due to constraint of time and resource. The other limitation is that this study is done only at single hospital; hence, further studies should be conducted in different hospitals of Addis Ababa to have findings representing the whole population.

## 5. Conclusion

This study has revealed that the prevalence of anemia in pregnancy was low (11.62%) compared to the findings of other areas of Ethiopia. Gestational age (trimester) and iron/folic acid supplementation were statistically associated with anemia in this study. Therefore, iron supplementation and health education to create awareness about the importance of early booking for antenatal care are recommended to reduce anemia.

## Figures and Tables

**Table 1:** Sociodemographic, obstetric, and other characteristics of pregnant women (N=284).

**Variables**	**Frequency**	**Percentage (**%**)**
**Age group (years)**		
≤20	16	5.6
21-25	93	32.7
26-30	118	41.5
31-35	43	15.1
≥36	14	4.9

**weight group (kg)**		
40-49	28	9.9
50-59	89	31.3
60-69	102	35.9
70-79	40	14.1
≥80	25	8.8

**Occupation**		
Farmer	16	5.6
Housewife	164	57.7
Government	24	8.5
Student	8	2.8
Private	72	25.4

**Educational status**		
Illiterate	42	14.8
Elementary	115	40.5
Secondary	54	19.0
Preparatory	23	8.1
University/college	50	17.6

**Residence**		
Rural	23	8.1
Urban	261	91.9

**Trimester**		
1st trimester	48	16.9
2nd trimester	66	23.2
3rd trimester	170	59.9

**Previous Pregnancy**		
No	90	31.7
Yes	194	68.3

**Gravidity**		
1	90	31.7
2-4	166	58.5
≥5	28	9.9

**Number of child**		
None	124	43.7
1	85	29.9
2	51	18.0
≥3	24	8.5

**Space b/n the current pregnancy and the last child**		
<1 year	122	43.0
1 year	12	4.2
2 year	21	7.4
3 year	33	11.6
4 year and above	96	33.8

**Blood loss**		
No	256	90.1
Yes	28	9.9

**Abortion**		
No	207	72.9
Yes	77	27.1

**Number of abortion**		
None	207	72.9
Once	56	19.7
Two and above	21	7.4

**Iron/folic acid Supplementation**		
No	117	41.2
Yes	167	58.8

**Meat and animal product**		
No	12	4.2
Yes	272	95.8

**Frequency of eating meat and animal product**		
None	12	4.2
every day	37	13.0
every 2 days	22	7.7
once in week	160	56.3
once in month	53	18.7

**Fruit and Vegetable**		
No	3	1.1
Yes	281	98.9

**Frequency of eating fruit and vegetable**		
None	3	1.1
every day	100	35.2
every 2 days	58	20.4
once in week	120	42.3
once in month	3	1.1

**Table 2:** Prevalence of anemia among pregnant women by sociodemographic, obstetric, and other characteristics of pregnant women (N=284).

**Variables**	** Anemia status**		**AOR (95**%** CI)**	**P value **
	**Non-Anemic (**%**)**	**Anemic (**%**)**		
**Age group**				
≤20	15 (93.8%)	1 (6.2%)	1	
21-25	86 (92.5%)	7 (7.5%)	1.53 (.16-14.70)	0.71
26-30	100 (84.7%)	18 (15.3%)	4.47 (.47-42.83)	0.19
31-35	37 (86.0%)	6 (14.0%)	5.58 (.457-68.10)	0.18
≥36	13 (92.9%)	1 (7.1%)	3.55 (.13-100.17)	0.46

**weight group**				
40-49	27 (96.4%)	1 (3.6%)	1	
50-59	81 (91.0%)	8 (9.0%)	2.34 (.25-21.85)	0.45
60-69	84 (82.4%)	18 (17.6%)	5.14 (.57-46.17)	0.14
70-79	36 (90.0%)	4 (10.0%)	1.38 (.12-16.30)	0.80
≥80	23 (92.0%)	2 (8.0%)	1.47 (.101-21.47)	0.78

**Occupation**				
Farmer	16 (100.0%)	0 (.0%)		
Housewife	141 (86.0%)	23 (14.0%)		
Government	22 (91.7%)	2 (8.3%)		
Student	7 (87.5%)	1 (12.5%)		
Private	65 (90.3%)	7 (9.7%)		

**Educational status**				
Not educated	40 (95.2%)	2(4.8%)		
Elementary	98(85.2%)	17 (14.8%)		
Secondary	48(88.9%)	6(11.1%)		
Preparatory	21 (91.3%)	2(8.7%)		
University/college	44 (88.0%)	6 (12.0%)		

**Residence**				
Rural	23 (100.0%)	0 (.0%)		
Urban	228 (87.4%)	33 (12.6%)		

**Trimester**				
1st trimester	46 (95.8%)	2(4.2%)	1	
2nd trimester	55(83.3%)	11(16.7%)	6.72 (1.17-38.45)	0.03^*∗∗*^
3rd trimester	150 (88.2%)	20(11.8%)	8.31 (1.24-55.45)	0.03^*∗∗*^

**Previous Pregnancy**				
No	81 (90.0%)	9 (10.0%)		
Yes	170(87.6%)	24(12.4%)		

**Gravidity**				
1	81 (90.0%)	9 (10.0%)		
2-4	145 (87.3%)	21 (12.7%)		
≥5	25 (89.3%)	3 (10.7%)		

**Number of child**				
None	112 (90.3%)	12 (9.7%)	1	
1	70 (82.4%)	15 (17.6%)	2.49 (.000)	0.99
2	47 (92.2%)	4 (7.8%)	6.81 (.000)	0.99
≥3	22 (91.7%)	2 (8.3%)	6.99 (.000)	0.99

**Space b/n the current pregnancy and the last child**				
0 year	110(90.2%)	12(9.8%)	1	
1 year	11(91.7%)	1(8.3%)	.00(.00)	0.99
2 year	20(95.2%)	1 (4.8%)	.00(.00)	0.99
3 year	30(90.9%)	3(9.1%)	.00(.00)	0.99
4 year and above	80 (83.3%)	16 (16.7%)	.00(.00)	0.99

**Blood loss**				
No	224(87.5%)	32(12.5%)	1	
Yes	27 (96.4%)	1(3.6%)	.29 (.03-2.52)	0.26

**Abortion**				
No	185(89.4%)	22 (10.6%)	1	
Yes	66 (85.7%)	11(14.3%)	2.06 (.82-5.15)	0.12

**Number of abortion**				
None	185 (89.4%)	22 (10.6%)		
Once	48 (85.7%)	8 (14.3%)		
Two and above	18 (85.7%)	3 (14.3%)		

**Iron/folic acid Supplementation**				
No	100 (85.5%)	17(14.5%)	4.03(1.49-10.92)	0.01*∗∗*
Yes	151 (90.4%)	16 (9.6%)	1	

**Meat and Animal Product**				
No	11(84.6%)	2(15.4%)		
Yes	240 (88.6%)	31(11.4%)		

**Frequency of eating meat and animal product**				
every day	33 (89.2%)	4(10.8%)		
every 2 day	19(86.4%)	3(13.6%)		
once in week	139 (86.9%)	21 (13.1%)		
once in month	50 (94.3%)	3 (5.7%)		
none	10 (83.3%)	2 (16.7%)		

**Fruit and Vegetable**				
No	2 (66.7%)	1 (33.3%)		
Yes	249 (88.6%)	32 (11.4%)		

**Frequency of eating fruit and vegetable**				
every day	89 (89.0%)	11 (11.0%)		
every 2 day	51 (87.9%)	7 (12.1%)		
once in week	106 (88.3%)	14 (11.7%)		
once in month	3(100.0%)	0 (.0%)		
none	2 (66.7%)	1 (33.3%)		

^*∗∗*^P < 0.05 (statistically significant association) for the Adjusted Odds Ratio (AOR).

## Data Availability

The data of this study cannot be shared publicly due to presence of sensitive (confidential) participants' information and additional data compared to that used in this publication. But the data are available from the corresponding author on reasonable request.

## List of Abbreviations

ANC: Antenatal care
EDTA: Ethylenediaminetetraacetic acid
Hb: Hemoglobin
MDGs: Millennium Development Goals
RBC: Red blood cell
SPHMMC: St.Paul'sHospital MillenniumMedicalCollege
WHO: World Health Organization.
